# State-of-the-Art Review on the Treatment of Axial Spondyloarthritis

**DOI:** 10.3390/medsci13010032

**Published:** 2025-03-16

**Authors:** Evripidis Kaltsonoudis, Panagiota Karagianni, Tereza Memi, Eleftherios Pelechas

**Affiliations:** 1 Department of Rheumatology, Chatzikosta General Hospital, 454 45 Ioannina, Greece; pelechas@doctors.org.uk; 2 Medical School, Department of Microbiology, University of Ioannina, 451 10 Ioannina, Greece; karagiannigiota82@gmail.com; 3 Medical School, Department of Rheumatology, University of Ioannina, 451 10 Ioannina, Greece; teresamemi@libero.it

**Keywords:** axial spondyloarthritis, JAK inhibitors, TNF inhibitors, IL-17, IL-23

## Abstract

The term axial spondyloarthritis (axSpA) encompasses patients with both radiographic (r-axSpA) and non-radiographic (nr-axSpA) forms of the disease. These are two entities within the same family that share many genetic and pathogenic factors, but they also have significant differences. For example, the male-to-female ratio is 2:1 in r-axSpA and 1:1 in nr-axSpA. Additionally, the prevalence of the HLA-B27 gene is notably higher in r-axSpA. Early diagnosis remains an unmet need, with magnetic resonance imaging (MRI) being the most important tool for diagnosis and disease monitoring. Early detection is crucial, as it allows for timely treatment, increasing the chances of preventing new bone formation and long-term structural bone damage. Various cytokines, such as tumor necrosis factor (TNF)-α and interleukin-17, play active roles in the disease’s pathogenesis, although the exact mechanisms of interaction are not yet fully understood. Clarifying these mechanisms will be key to developing new classification criteria, screening methods, and more personalized, targeted therapies. Non-steroidal anti-inflammatory drugs (NSAIDs), TNF inhibitors, interleukin-17 blockers, and, more recently, Janus kinase (JAK) inhibitors, are the most effective treatments for both radiographic and non-radiographic axial spondyloarthritis.

## 1. Introduction

Axial spondyloarthritis (axSpA) encompasses the full spectrum of patients with and without radiographic sacroiliitis. There is substantial evidence supporting the use of the term “axSpA”, which is why it has been adopted in revised guidelines [1,2].

With advancements in distinguishing between radiographic (r-axSpA) and non-radiographic axSpA (nr-axSpA), as well as understanding the various immunopathogenic mechanisms linking inflammation to new bone formation, the diagnosis and management of axSpA have significantly improved over the past twenty-five years. Greater insight into the disease’s pathophysiology and the identification of various pathogenic pathways, along with the involvement of both old and new pro-inflammatory cytokines, have led to the development of new therapies that closely approach the therapeutic goal of treat-to-target. These therapies have resulted in significant improvements in disease symptoms and patient quality of life [3,4].

The revised classification criteria for axSpA include patients with and without evident radiographic sacroiliitis and have greatly contributed to earlier diagnosis and timely initiation of treatment [5]. However, despite these advances, the diagnosis of axSpA still faces significant delays, as evidenced by numerous studies. These delays vary widely between countries, and although referral times have improved, the time from symptom onset to diagnosis remains a concerning point.

In terms of treatment, the management of axSpA includes both non-pharmacological and pharmacological interventions. Beyond exercise and physical therapy, which have been known for their benefits for centuries [6,7,8], the four major categories of approved therapeutic options include NSAIDs (non-steroidal anti-inflammatory drugs-available since the 1950s), TNF inhibitors (FDA-approved in 2003), IL-(interleukin) 17 inhibitors (FDA-approved in 2016), and, more recently, Janus kinase (JAK) inhibitors (FDA-approved) [9].

As part of the management algorithm, NSAIDs remain the first-line pharmacological treatment alongside physical therapy. TNF-α inhibitors, IL-17 inhibitors, and more recently JAK inhibitors, are available as second-line treatments. All these agents have demonstrated efficacy in improving the signs and symptoms of the disease [10].

Moreover, there have been several new developments in axSpA, including updated treatment guidelines [11] and new data on the impact of biological (b) DMARDs (disease modifying anti-rheumatic drugs) on the structural progression of axSpA. Progressive spinal ankylosis is a daunting aspect of axSpA, as it leads to loss of mobility and disability due to the structural progression of the disease [12]. Despite the rapid and sustained anti-inflammatory effects observed with MRI (magnetic resonance imaging) and inflammatory markers in patients in remission with the use of various b-DMARDs and NSAIDs, the beneficial impact of these treatments on radiographic progression has not yet been proven in axSpA. Long-term follow-up data suggest that successful inflammation control may slow radiographic progression, particularly with newer agents, but these results require further evaluation [13].

Therefore, more information is needed about new therapeutic molecular targets involved in the inflammation and ossification processes. Additionally, in recent years, strategic trials have been conducted to reduce or discontinue TNF inhibitors in patients in remission. However, withdrawing TNF inhibitors often leads to a relapse in disease activity in axSpA [14].

Further strategic treat-to-target studies in axSpA have been conducted, aiming for more effective and holistic disease management [2,15,16]. Additionally, the discovery that IL-23 blocking agents were ineffective in axSpA was somewhat surprising, and the possible underlying mechanisms for this lack of response are being discussed and investigated [17].

Given these findings, this review aims to explore recent advancements in the management of axSpA, with a focus on the latest data and promising therapies that improve disease outcomes.

## 2. Methods

This review was conducted by systematically analyzing peer-reviewed literature related to axSpA, focusing on recent advancements in diagnosis, classification criteria, and treatment strategies. The sources included clinical trials, meta-analyses, systematic reviews, and consensus statements published in major rheumatology journals.

A comprehensive search was performed using databases such as PubMed, Embase, and Scopus, covering articles published from 2000 to the present. Keywords included “axial spondyloarthritis”, “radiographic and non-radiographic axSpA”, “TNF inhibitors”, IL-17 inhibitors”, “NSAIDs”, “JAK inhibitors”, “treatment guidelines”, and “disease progression”. References were selected based on their relevance, study design, and contribution to understanding axSpA’ pathophysiology and management.

The review incorporated data from over 40 clinical trials, 28 systematic reviews, and numerous real-world observational studies, including studies on rehabilitation and non-pharmacological interventions. The decision to include certain studies was based on their sample size, study duration, and level of evidence, with preference given to randomized controlled trials and longitudinal studies.

Additionally, major guidelines and recommendations from the Assessment of Spondyloarthritis International Society, European Alliance of Associations for Rheumatology, and American College of Rheumatology were evaluated to provide a comprehensive view of current treatment paradigms.

The objective of this review is to synthesize the latest evidence on axSpA treatments, highlight ongoing challenges, and discuss emerging therapeutic strategies, with a particular focus on disease-modifying agents and their impact on structural progression.

### 2.1. Physical Therapy and Exercise in axSPA

Axial spondyloarthritis is an inflammatory disease primarily affecting the axial skeleton. It leads to varying degrees of spinal mobility restriction, stiffness, pain, and loss of functional capacity. Rehabilitation, particularly through physical therapy and exercise, is considered an important part of managing the disease, often in conjunction with appropriate medication. Various rehabilitation methods have been applied and are available to support individuals with axSpA, but there is no established protocol outlining the sequence of these methods based on current literature [7,18].

A comprehensive review of 28 studies involving a total of 1926 axSpA patients demonstrated that those who participated in exercise programs experienced significant benefits compared to those who did not. The review concluded that patient education, active participation, and motivation played crucial roles in the overall treatment outcomes. Based on this review, a four-phase sequential rehabilitation protocol has been established for the benefit of axSpA patients [19].

Overall, the findings indicate that a personalized home exercise program, whether supervised or not, is more effective than no intervention. Additionally, supervised group physical therapy is shown to be more beneficial than home exercises alone, and a combined approach of inpatient spa therapy followed by group physical therapy is more effective than group physical therapy alone [20].

### 2.2. Non-Steroidal Anti-Inflammatory Drugs (NSAIDs)

According to recently published updated treatment recommendations for axSpA [21], the treatment algorithm emphasizes early intervention with NSAIDs in combination with physical therapy and exercise. Evidence supporting the effectiveness of physical therapy was reviewed in a systematic analysis, which found that regular exercise improved disease activity, pain, functionality, and spinal mobility, though the study sample sizes were small [22].

However, no definitive conclusions have been established regarding the role of exercise in preventing the development of syndesmophytes in the spine. Therefore, NSAIDs are globally recommended as the first-line treatment for axSpA, whether combined with exercise and physical therapy or not [11].

NSAIDs appear to be more effective when prescribed early in the disease course, with one study showing that up to 35% of patients achieved significant improvement within four weeks [23]. Additionally, evidence suggests that NSAIDs effectively manage the signs and symptoms of axSpA, although moderate to low-quality evidence indicates that long-term ossification progression may not differ significantly from placebo. Some studies suggest that continuous NSAID use may reduce radiographic spinal progression, but these findings require further confirmation [24,25].

Until more data are available, NSAIDs should be recommended primarily for treating the signs and symptoms of axSpA, rather than for preventing the development of syndesmophytes, which can lead to structural damage and severe, irreversible disability [26].

### 2.3. Glucocorticoids and Conventional Synthetic DMARDs (csDMARDs)

Chronic administration of glucocorticoids (GCs) should generally be avoided because the high doses required for disease control are associated with significant long-term toxicity and morbidity [27]. Conventional synthetic (cs)DMARDs, such as leflunomide, sulfasalazine, and hydroxychloroquine, do not appear to be effective in treating axial disease [11].

Furthermore, the combination of methotrexate with anti-TNF agents has not led to better therapeutic outcomes, although it is noteworthy that co-administration with infliximab reduces the development of antibodies against the drug. On the other hand, in cases of uveitis, which sometimes coexists with axSpA, anti-TNF-based therapies have demonstrated good clinical responses, with better safety profiles observed in the combination of etanercept and methotrexate compared to the other two anti-TNF monoclonal antibodies combined with methotrexate [28]. Moreover, etanercept does not have documented efficacy in eye and bowel involvement in axSpA, so adding methotrexate in these cases could be particularly beneficial [29].

### 2.4. When to Initiate Biological Therapy in axSpA

Studies involving various approved biological drugs for both r-axSpA and nr-axSpA indicate that higher rates of achieving therapeutic goals are attained when these treatments are used in the early stages of the disease. This suggests that a change in prescribing behavior could lead to a greater likelihood of achieving remission, which is the primary and often realistic goal of biological therapy [2,30].

Accordingly, based on extensive previous data, a patient diagnosed with r-axSpA or nr-axSpA who has failed standard treatment (more specifically, two NSAIDs for at least four weeks) and presents with proven inflammation through measurable indicators, such as ASDAS ≥ 2.1, BASDAI ≥ 4, or signs of activity like bone edema on MRI or elevated and persistent CRP, should be evaluated for the initiation of a bDMARD [26].

Additionally, the clear selection of ASDAS as the instrument used to determine a patient’s eligibility for treatment with b/ts (targeted synthetic) DMARDs (ASDAS ≥ 2.1), as well as for treatment continuation (improvement ≥ 1.1), represents an important innovation in these recommendations [2]. All findings underscore the correlation between active inflammation and timely intervention with a biological agent, as well as the value of repeated evaluations during routine treatments, such as NSAIDs, exercise, and physiotherapy.

### 2.5. TNF-α Inhibitors

The introduction of TNF inhibitors (TNFi) in the treatment of axSpA marked a new era in disease management. TNFi, such as adalimumab, certolizumab, etanercept, golimumab, and infliximab, are approved for use in r-axSpA in Europe and the USA. Moderate to high-quality evidence supports a clinically significant benefit compared to placebo in improving disease activity and functionality, as well as achieving partial remission within a relatively short period after initiating treatment [31].

Recent long-term follow-up data of patients with axSpA who received TNFi therapy show a slow but detectable radiographic progression of the disease [21,32]. In another study, Kampman et al. demonstrated that patients with axSpA treated with anti-TNF exhibited linear disease progression within the first four years [33].

A systematic review and meta-analysis of literature data indicated that TNFi agents exert a protective effect on the progression of radiographic spinal damage, and they should be administered for four or more years. However, this is not definitive, as the authors themselves highlight the need for additional studies to confirm or refute these findings and to definitively answer the important question regarding radiographic progression with the use of these drugs [34].

The best predictive factors for a good response to TNFi in patients with r-axSpA include an elevated C-reactive protein (CRP), shorter duration of symptoms or younger age, and active inflammation confirmed by MRI. Conversely, in patients with nr-axSpA, MRI assessments have low sensitivity and specificity, structural progression is slower and rarer, and central pain is more common in this patient group [35,36,37,38,39,40].

Discontinuation of TNFi has been unsuccessful, as it leads to more frequent relapses compared to continuing therapy. However, TNFi tapering has shown promising results in patients with prolonged disease remission [11,41], as it has been examined in other rheumatic diseases [42]. Therefore, and without significant risk, cautious attempts should be made to reduce TNFi in cases of prolonged symptomatic control in axSpA patients, considering that the disease begins early in adulthood and treatment is lifelong.

Additionally, monoclonal antibodies against TNF have been associated with a reduced risk of uveitis compared to the soluble TNF receptor protein (etanercept) and IL-17i (secukinumab) [2].

From 2003 to 2016, TNFi dominated the landscape of biological therapy for axSpA. During this period, research leading to the discovery of the IL-23/IL-17 axis pathway unveiled new therapeutic targets based on the disease’s pathophysiology, and new agents targeting molecules in this pathway were developed, demonstrating proven efficacy in various outcomes of the inflammatory process. Nevertheless, experience with TNFi in terms of safety and efficacy, as well as broad coverage of extra-skeletal manifestations, such as eye and bowel involvement in axSpA, significantly surpasses that of other cytokine inhibitors both in case volume and monitoring duration. For these reasons, current practice and treatment guidelines place TNFi high in the therapeutic algorithm.

### 2.6. Interleukin-17 Inhibitors (IL-17i)

IL-17A is a member of the IL-17 cytokine superfamily, which includes IL-17A, IL-17B, IL-17C, IL-17D, IL-17E, and IL-17F [43]. Inhibition of IL-17 can be achieved either by targeting IL-17A with the two approved molecules, secukinumab and ixekizumab, or through dual blockade of IL-17A and IL-17F with bimekizumab.

Current systematic literature reviews confirm the efficacy and safety of IL-17i (particularly secukinumab and ixekizumab) in patients with axSpA [44]. Several large and high-quality trials have demonstrated the effectiveness and safety of secukinumab and ixekizumab across both spectra of axSpA (nr-axSpA and r-axSpA). Furthermore, both drugs have been evaluated for their efficacy in patients who were TNFi-naïve or had an inadequate response to TNFi [44].

Beyond the high efficacy in naïve axSpA patients, administering IL-17 inhibitors to those with prior exposure to TNFi agents resulted in higher response rates compared to patients receiving placebo [45,46].

The effect of secukinumab on new bone formation, indicating radiographic disease progression, was examined in a single phase III RCT (ΔmSASSS, W104 [253, 0.31 ± 1.94, 0.54 ± 2.45, P = NS]). The study suggested that IL-17 inhibition may reduce the progression of new bone formation. However, the two-year timeframe is short for evaluating structural changes, and longitudinal analyses of these results are required. Until more robust data become available, the reduction in new bone formation with IL-17i use in axSpA remains uncertain [26,47]. Data on ixekizumab’s ability to inhibit structural progression are currently unavailable.

Several new molecules targeting various IL-17 receptors are showing promising results in phase II and III trials. Bimekizumab, a monoclonal IgG1 antibody that selectively inhibits interleukin (IL)-17F in addition to IL-17A, has been investigated for its efficacy and safety across the axSpA spectrum. Results from two parallel phase III randomized controlled trials indicate that dual inhibition of IL-17A and IL-17F with bimekizumab led to significant and rapid improvements compared to placebo and was well-tolerated in patients with nr-axSpA and r-axSpA [48,49].

### 2.7. Janus Kinase Inhibitors (JAKi)

The JAK-STAT pathways have also been explored in phase III trials for axSpA. JAK inhibition involves specific JAK-STAT pathways. For instance, inhibition of JAK1 can interfere with the signaling of interferons α, β, and γ, as well as cytokines IL-6 and IL-22, potentially affecting Th1 and Th17 responses in axSpA [50]. Additionally, JAK3 inhibition may lead to reduced IL-7 production, which can interfere with the differentiation and function of innate lymphoid cells, important factors in maintaining homeostasis in this disorder [51].

Agents targeting the TYK2 kinase, significantly involved in the IL-23/IL-17 pathway signaling, could be effective, although current research data show that targeting IL-23 has not yielded the desired results and remains questionable [52,53].

JAKi represent an entirely new approved class of drugs for r-axSpA. To date, proven efficacy has been demonstrated by tofacitinib and upadacitinib in r-axSpA [54]. Due to their relatively recent approval, these agents lack long-term safety data compared to TNFi and even IL-17 inhibitors. This factor, combined with safety warnings from surveillance studies in rheumatoid arthritis and high costs, makes this therapy less flexible and should be prescribed cautiously and judiciously.

### 2.8. Interleukin-12/23 and IL-23 Inhibitors

**Figure 1:**
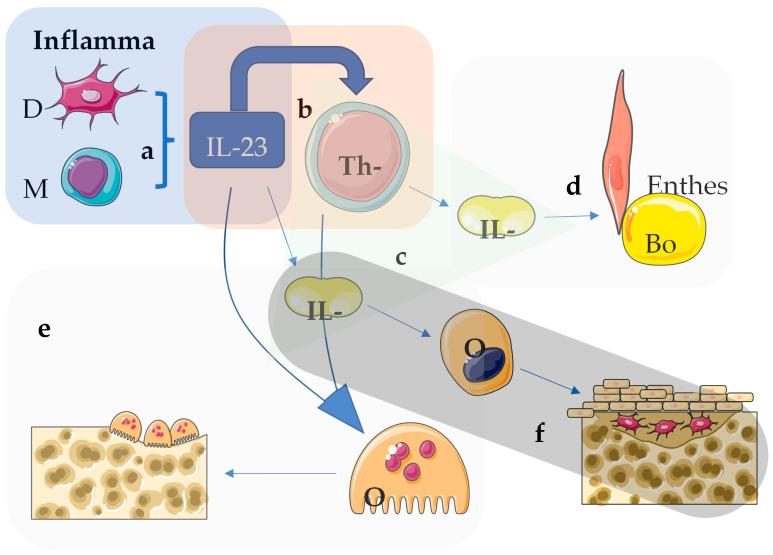
The implication of IL-23 and IL-17 in AxSpA. In AxSpA, IL-23 plays a central role in promoting inflammation, particularly with the axial skeleton (spine and sacroiliac joints). IL-23 is produced primarily by dendritic cells and macrophages in response to inflammation or tissue damage (**a**). In AxSpA, an overproduction of IL-23 occurs, which stimulates immune cells, particularly Th17 cells (**b**). These Th17 cells are crucial mediators of inflammation. Th17 cells produce another important cytokine, IL-17 (**c**). IL-17 acts on various tissues, particularly at sites like the entheses, leading to the development of enthesitis (**d**), a hallmark of AxSpA, causing pain and stiffness. IL-23 indirectly contributes to the dysregulated balance between bone erosion and bone formation seen in AxSpA. Inflammatory cells activated by IL-23 and IL-17 can trigger the activity of osteoclasts, leading to bone erosion at the affected joints (**e**). Conversely, chronic inflammation in AxSpA is also associated with abnormal new bone formation (syndesmophytes) in the spine, contributing to structural damage and spinal fusion over time (**f**).

IL-23 is a pleiotropic cytokine crucial for the differentiation, survival, and expansion of conventional (αβ) T cells and unconventional (γδ) T cells, thereby influencing numerous immune responses [55]. Through the IL-23/IL-17 axis, IL-23 significantly modulates IL-17 production (Figure 1). Based on these findings and the central role of IL-23 in axial disease in experimental spondyloarthritis models, inhibiting this cytokine was expected to be an effective treatment strategy for axSpA.

While initial results from a small study using IL-12/23 inhibition (ustekinumab) were encouraging [56], a phase III RCT failed to show significant effects on disease symptoms and reduction in inflammatory burden in r-axSpA, leading to its termination [57]. Despite the close collaboration between IL-23 and IL-17 cytokines, IL-17 can be produced by many cell types independently of IL-23. Another reason could be that IL-23 plays a key role in the disease’s initiation during the preclinical or early phase of axSpA but may not be necessary for the continuation and maintenance of inflammation [58].

These factors may explain why IL-12/23 inhibition failed to demonstrate effectiveness in multiple large trials in r-axSpA. The primary endpoint based on the T2T strategy was not achieved, although improvements were observed in secondary outcomes. This led to a trial targeting IL-23 in axSpA with risankizumab (18 mg, 90 mg, 180 mg) in a phase III RCT, which also proved ineffective [59].

The unexpected ineffectiveness of these two studies has somewhat questioned the role of the IL-23/IL-17 axis in the pathogenesis of SpA. Nonetheless, the IL-23/IL-17 axis in inflammation continues to be investigated and is rapidly evolving.

### 2.9. Unmet Needs

Despite progress in understanding the pathogenesis and treatment of axSpA, significant challenges remain:

- **Suboptimal Treatment Response:** A considerable number of patients do not achieve ideal therapeutic responses.
- **Limited Drug Options:** Available medications are fewer compared to other conditions like rheumatoid arthritis.
- **Lack of Biomarkers:** There is an absence of biomarkers to identify early which patients with chronic back pain are at increased risk of progressing to axial spondyloarthritis.
- **Predictive Indicators:** Predictors for treatment response, indicating the most suitable drug, are lacking.
- **Extra-Skeletal Manifestations:** Determining which patients will develop extra-skeletal manifestations remains challenging.
- **Non-Inflammatory Pain Mechanisms:** Understanding and addressing mechanisms underlying non-inflammatory pain (e.g., depression, anxiety) are needed.
- **Delayed Diagnosis:** Strategies to improve delayed diagnosis are still required.

## 3. Discussion

In Table 1, all the available treatments are presented. Despite the increasing number of therapeutic options available for axSpA patients, several challenges remain unmet [60]. Approximately half of the patients participating in clinical studies do not achieve significant differences in primary endpoints. This can partly be explained by the fact that the efficacy observed with IL-17 inhibition and JAK inhibition is largely similar and comparable to that seen with TNFi agents. Consequently, there are no clear guidelines for first-line therapy choice, leaving decisions dependent on the physician’s experience, knowledge, and, in some cases, bias. Another hindrance is the high cost and, in some cases, limited access to modern therapeutic regimens.

Recommendations from major organizations like ASAS-EULAR for managing axSpA, covering both nr-axSpA and r-axSpA, include both non-pharmacological and pharmacological approaches. The increasing availability of biological agents targeting different cytokines and the lack of relevant head-to-head trials comparing these molecules in axSpA result in a lack of guidelines regarding their administration sequence. Thus, beyond recommendations favoring monoclonal antibodies against TNF in patients with a history of recurrent uveitis or active IBD and preferring an IL-17i in patients with significant psoriasis, the choice of therapeutic agent is empirical [44,54,61].

Safety data for IL-17i and JAKi are available only from RCTs [44,54]. Conversely, extensive experience with TNFi in safety matters significantly surpasses that of other cytokine and kinase inhibitors both in case volume and monitoring duration.

Moreover, newer drugs on the market are considerably more expensive than previous biologicals, which should be considered when prescribing, especially since, to date, head-to-head studies in axSpA have not demonstrated any difference in efficacy (if any exists) that would justify a much more expensive drug choice [62].

Considering that overweight and obese patients with inflammatory arthritis have higher disease activity and lower chances of achieving and maintaining treatment goals, it has been shown that weight/BMI should be considered in the treatment plan for patients with inflammatory arthritis, with its impact being more pronounced for TNFi compared to other b/tsDMARDs [63]. Thus, although BMI seems generally related to treatment response and not only to TNFi, data from this Greek study suggest that TNFi agents might be placed lower in the preference order for a biological agent.

Finally, since pain is a significant component of axSpA, comorbidities such as depression and sleep disorders should be promptly recognized and corrected, both as part of a holistic therapeutic approach and because they can lead to confusing conclusions regarding the effectiveness of agents targeting the disease’s inflammatory components [64].

## 4. Conclusions

Significant strides have been made in managing patients with axSpA over the past decade. Improved understanding of the disease’s pathophysiology has led to the introduction of newer (IL-6 inhibitors) and novel (JAK inhibitors) drugs that substantially contribute to reducing the therapeutic gap left by TNFi. Unexpected failure of IL-23 inhibitors in achieving endpoints has been observed, with causes remaining unclear and requiring further investigation. Research also focuses on whether existing therapies inhibit or merely slow radiographic progression, with no definitive answer yet available. In patients with prolonged remission, spacing out dosing intervals is recommended, but not discontinuation of medications. According to guidelines, there is no clear directive for preferring a particular agent after failure of standard therapy (NSAIDs, exercise, and physiotherapy). However, comorbidities, extra-skeletal manifestations, safety, experience, broad coverage of manifestations, and cost should certainly be taken into account.

### Future Directions

Future efforts in managing axSpA should focus on optimizing personalized treatment approaches. With the advent of precision medicine, biomarkers predictive of treatment response and disease progression need further exploration to guide therapeutic decisions. Head-to-head trials comparing bDMARDs and tsDMARDs will help establish evidence-based treatment sequences, addressing the current approach. Additionally, research into the role of comorbidities, such as obesity, depression, and sleep disorders, in treatment efficacy could refine holistic care strategies. The continuous development of biosimilars may further improve global accessibility, particularly in resource-limited settings. Finally, exploring optimal dosing strategies and de-escalation protocols in remission could further enhance quality of life and reduce treatment burden for axSpA patients.

## Figures and Tables

**Table 1:** Available treatments for r-axSpA and nr-axSpA.

Category of Treatment	Drug Class	Example Medications	Mechanism of Action	Approved for r-axSpA	Approved for nr-axSpA
NSAIDs	NSAIDs	Ibuprofen, Naproxen, Diclofenac, Etoricoxib	Inhibition of COX-1 and COX-2 enzymes, reducing inflammation and pain	YES	YES
Glucocorticoids	Steroids	Prednisone, Methylprednisolone	Reduction in inflammation and suppression of the immune system	Conditionally, depending on disease’s phenotype; not for axial disease	Conditionally, depending on disease’s phenotype; not for axial disease
DMARDs	Conventional synthetic DMARDs	Methotrexate, Sulfasalazine, Leflunomide	Reduction in inflammation and modulation of the immune response	Conditionally, depending on disease’s phenotype; not for axial disease	Conditionally, depending on disease’s phenotype; not for axial disease
Biologic DMARDs	TNF inhibitors	Adalimumab	Inhibition of TNF, a cytokine involved in inflammation	YES	YES
		Etanercept		YES	YES
		Infliximab		YES	NO
		Certolizumab		YES	YES
		Golimumab		YES	YES
	IL-17 inhibitors	Secukinumab	Inhibition of IL-17, a cytokine involved in inflammationBimekizumab inhibits IL-17A and F	YES	YES
		Ixekizumab		YES	YES
		Bimekizumab		YES	YES
Targeted synthetic DMARDs	JAK inhibitors	Tofacitinib		YES	NO
		Upadacitinib		YES	YES
Physical Therapy and Exercise	Physical Therapy Exercises	Stretching, Strengthening exercises	Improvement in mobility and reduction in pain through increased flexibility and muscle strengthening		

NSAIDs: non-steroidal anti-inflammatory drugs; COX: cyclooxygenase; DMARDs: disease-modifying anti-rheumatic drugs; JAK: Janus Kinase; IL: interleukin.

## Data Availability

No new data were created or analyzed in this study. Data sharing is not applicable to this article.
